# Aquaporins in the wild: natural genetic diversity and selective pressure in the PIP gene family in five Neotropical tree species

**DOI:** 10.1186/1471-2148-10-202

**Published:** 2010-06-29

**Authors:** Delphine Audigeos, Anna Buonamici, Laurent Belkadi, Paul Rymer, David Boshier, Caroline Scotti-Saintagne, Giovanni G Vendramin, Ivan Scotti

**Affiliations:** 1 INRA UMR 0745 EcoFoG (« Ecologie des forêts de Guyane »), Campus Agronomique, BP709 - 97387 Kourou, French Guiana, France; 2 Istituto di Genetica Vegetale, Sezione di Firenze, Consiglio Nazionale delle Ricerche, 50019 Sesto Fiorentino (Firenze), Italy; 3 Dept. of Plant Sciences, University of Oxford, South Parks Road, Oxford, OX1 3RB, UK

## Abstract

**Background:**

Tropical trees undergo severe stress through seasonal drought and flooding, and the ability of these species to respond may be a major factor in their survival in tropical ecosystems, particularly in relation to global climate change. Aquaporins are involved in the regulation of water flow and have been shown to be involved in drought response; they may therefore play a major adaptive role in these species. We describe genetic diversity in the PIP sub-family of the widespread gene family of Aquaporins in five Neotropical tree species covering four botanical families.

**Results:**

PIP Aquaporin subfamily genes were isolated, and their DNA sequence polymorphisms characterised in natural populations. Sequence data were analysed with statistical tests of standard neutral equilibrium and demographic scenarios simulated to compare with the observed results. Chloroplast SSRs were also used to test demographic transitions. Most gene fragments are highly polymorphic and display signatures of balancing selection or bottlenecks; chloroplast SSR markers have significant statistics that do not conform to expectations for population bottlenecks. Although not incompatible with a purely demographic scenario, the combination of all tests tends to favour a selective interpretation of extant gene diversity.

**Conclusions:**

Tropical tree PIP genes may generally undergo balancing selection, which may maintain high levels of genetic diversity at these loci. Genetic variation at PIP genes may represent a response to variable environmental conditions.

## Background

Within the tropics water availability, with soil fertility, is one of the most important environmental factors determining tree species richness [1] and distribution [2]. Although wet tropical regions are characterized by high annual rainfall, seasonality makes it unevenly distributed across the year such that even tropical humid forest can experience seasonal soil drought [3]. Regions currently occupied by luxuriant rainforest have also undergone decade- to century-long drier spells in both recent and geological past [4]. Tree species' natural range may have changed during those periods, but selective pressure may also have acted on extant populations. Genetic mechanisms of drought tolerance are therefore expected to have evolved in tropical tree species, and variation for these mechanisms is expected as different species, and populations within species, are adapted to different soil water availability optima. Study of potentially adaptive natural genetic diversity is needed to understand ecological mechanisms underlying the composition of such diverse ecosystems as tropical forests and to predict species responses to climate change (*e.g*. Amazonian forest ecosystems have been shown to be sensitive to damage induced by severe droughts [5]). Research on mechanisms and molecular bases of drought stress tolerance have been conducted for decades and several reviews exist [6-9], but only a few studies have focused on tropical trees as keystone species of tropical forests. There is a need therefore to explore the adaptive potential of forest tree populations [10] in natural tropical ecosystems.

The molecular basis of drought tolerance is extremely complex and a wide variety of expressional candidate genes has been suggested for example in *A. thaliana *[11] and in trees [12]. Among protein classes involved in response to drought, and the regulation of water balance in general, aquaporins are a good candidate starting point for the exploration of genetic diversity in natural populations of non-model species such as Neotropical rainforest trees, as they are ubiquitous, well known and the focus of in-depth functional studies in plants in general [13] and trees in particular [14]. In prokaryotes and eukaryotes, aquaporins play a channel role in water transport [14]. In plants, they form a large family divided into four subfamilies [15]. We chose for this study plasma membrane intrinsic proteins (PIPs), which are grouped in two subfamilies (PIP1 and PIP2). PIPs are well characterised and share a recent evolutionary history which permits quick isolation of multiple members of the gene family by homology-based methods. Moreover Alexandersson *et al*. [11] have shown that most PIP transcripts are down-regulated upon gradual drought stress, indicating that they are involved in, or affected by, response mechanisms to drought stress. Therefore, these genes may be under selection in natural populations. We tested the hypothesis that the genes coding for these proteins undergo natural selection in a set of Neotropical species.

The extent and selective/demographic meaning of diversity at drought-response candidate genes in natural populations of forest trees has been analysed in several recent studies (e.g. [16-25]). Some of these studies [17,21,23] present results on one or a few aquaporin genes, but no signature of selection or demography was detected at these loci when tested.

In this study we developed universal primers, based on plant sequences available in public databases, to sequence PIP genes in five tropical tree species: *Pachira quinata *(Bombacaceae), *Virola sebifera *(Myristicaceae), *Carapa guianensis *(Meliaceae), and two congeneric species *Eperua falcata *and *Eperua grandiflora *(Caesalpiniaceae). We describe their nucleotide diversity in natural populations and apply tests for departures from the standard neutral equilibrium to detect patterns that potentially indicate the action of natural selection. Our results point to the action of a combination of balancing selection and demographic events at these loci in populations of Neotropical forest trees.

## Results

**Figure 1:**
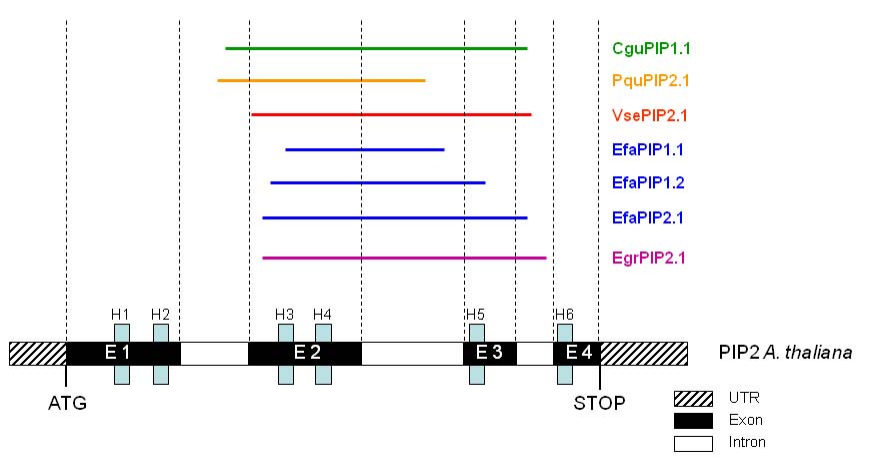
**Representation of the localisation of the different fragments compared to *Arabidopsis thaliana *PIP structure**. Blue boxes, located on the diagram of the Arabidopsis gene structure, represent the six transmembrane helices.

Amplifications with universal primers allowed the cloning of 1-5 different genes per species (Table 1). For eleven contigs, homology in TAIR databases [26] was found and sequence information was used for the development of gene-specific and species-specific primer pairs for the amplification of seven amplicons (Table 2). Successful amplification conditions were found for six primer pairs, while for one of the three *E. falcata*-specific primers transfer was possible to the congeneric species *E. grandiflora*. Using specific primers we sequenced a total of 4036 bp both in coding and non-coding regions (Figure 1). The identity of each amplicon as a different locus was proven at the population level by the detection of both homozygote and heterozygote individuals in the sampled populations. If there had been co-amplification of closely related isoforms, all individuals would have been expected to show heterozygosity for the sites differentiating the isoforms. As further evidence that single genes are amplified by each primer pair, non-synonymous polymorphisms, when occurring, mostly caused replacements between amino acids of similar structure and chemical properties, and in no case were stop-codon mutations detected. This led us to conclude that the amplicons correspond to separate, functional gene loci.

**Table 1:** Detailed results of isolation of PIPs gene fragments

			Universal primer-generated sequences							Specific-primer generated sequences		
			
Family	Genus	species	N_T_	Contigs	N_C_	TAIR	PIP subfamily	L_C _(bp)	Genbank	PCR Amplification	N	L (bp)
Meliaceae	*Carapa*	*guianensis*	46	CguContig1	11	NP_194071	PIP1	780	FJ709600	YES	70	673
				CguContig2	10	NP_200874	PIP2	782	FJ709601	Not tested	-	-
				CguContig3	6	NP_001078066	PIP1	747	FJ709602	Not tested	-	-

Bombacaceae	*Pachira*	*quinata*	46	PquContig1	16	NP_200874	PIP2	804	FJ709598	YES	32	513
				PquContig2	10	NP_195236	PIP2	628	FJ709599	NO	-	-

Myristicaceae	*Virola*	*sebifera*	22	VseContig1	22	NP_195236	PIP2	902	FJ807641	YES	46	627

Fabaceae	*Eperua*	*falcata*	96	EfaContig1	39	NP_182120	PIP1	484	FJ807642	YES	154	459
				EfaContig2	9	NP_181254	PIP2	485	FJ807643	NO	-	-
				EfaContig3	5	NP_181254	PIP2	554	FJ807644	NO	-	-
				EfaContig4	4	NP_200874	PIP2	553	FJ807645	YES	166	572
				EfaContig5	2	NP_171668	PIP1	545	FJ807646	YES	206	521

Legend: N_T_: number of sequenced PCR fragments amplified using universal degenerate primers; Contigs: CodonCodeAligner-generated contigs (putatively corresponding to separate genes, for each of which specific primers were designed); N_C_: number of sequences per contig; TAIR: closest BlastX match from the TAIR database; PIP subfamily: subfamily to which belongs the best TAIR match; L_C _: length of contig alignments; Genbank: Genbank/EMBL accession numbers; PCR amplification: presence/absence of a single PCR product using specific primers (YES = successful PCR, single PRC product; NO = multiple or no PCR products; not tested = primer pairs were developed but not used for the present study); L: length (bp) of the specific primer-generated PCR products

**Table 2:** Description of the PIPs fragments amplification conditions

	*Species*	*Primer Name*	*Primer sequences (5' → 3')*	*T_a _(°C)*	*Size (bp)*	*Genbank accession number*
a.	All	PIP2H2.2	F: CTYGTYTACTGCACHGCY	64	850	-
		PIP2H6.1	R: CCVACCCARAADATCCAN			
b.	*Carapa guianensis*	CguPIP1.1+0018	F: CGGCATTTCAGGTCATCTC	54	760	FJ709600
		CguPIP1.1-0780	R: CCAACCCAGAAAATCCAGTG			
	*Pachira quinata*	PquPIP2.1+0017	F: GCCGGTATCTCTGGTGAGTG	64	650	FJ709598
		PquPIP2.1-0672	R: CCACGCCTTCTCTTTGTTGT			
	*Virola sebifera*	VsePIP2.1+0032	F: CGCGTATCTCTCTCTTCAACG	59	750	FJ807641
		VsePIP2.1-0788	R: CACACGCACACACACAATG			
	*Eperua falcata*	EfaPIP1.1+0043	F: CCCAGCAGTGACCTTCG	64 → 57 ^(1)^	550	FJ807642
		EfaPIP1.1-0487	R: AACCAAGAACACAGCGAACC			
		EfaPIP1.2+0040	F: CAACCCGGCTGTGACC	64 → 57 ^(1)^	550	FJ807646
		EfaPIP1.2-0487	R: GCCAAATGGACCAAGAACAC			
		EfaPIP2.1+0034 ^(2)^	F: GCACATAAATCCGGCAGTG	64 → 57 ^(1)^	650	FJ807645
		EfaPIP2.1-0484 ^(2)^	R: CCGACCCAGAAGATCCAC			

Legend: Primer names, primer sequences, annealing temperature, size of the sequenced fragment, accession number of the closest *Arabidopsis thaliana *BLAST match. Primer names are derived from clone name followed by a sign corresponding to the direction of the synthesis ("+" for "forward", "-" for "reverse") and a number corresponding to the position of the primer's 5' end relative to the clone's sequence. ^(1) ^PCR touchdown: the first seven cycles with one degree decrease each cycle, from 64°C to 57°C. Other cycles at 57°C

^(2) ^For *E. grandiflora*, the same primers and conditions were used for PCR amplifications.

Polymorphism distribution in each gene fragment and each species is shown in table 3. Polymorphism was identified in all species, although the amount varied according to gene and species. A total of 79 SNPs (including indels) was found, distributed across coding and non-coding regions and between synonymous and non-synonymous sites. Details of the distribution of these polymorphisms in coding and non-coding regions, and between synonymous and non-synonymous sites within the latter, are provided in Table 3 (note that the total number of SNPs in Table 3 sums to more than 79 because they are reported at the population level, not the species level, for the species for which two populations have been analysed).

**Table 3:** Genetic diversity and results of mutation-drift equilibrium tests for each gene

*Genes [populations]*	*N*	*L*	*S*	*S_I_*	*S_S_*	*S_A_*	*A*	*H_d_*	***θ_π_***	***θ_*w*_***	rho	*D*		D*		F*		*F_S_*	
CguPIP1.1 [NW]	58	673	0	0	0	0	1	0	0	0	-	-		-		-		-	
CguPIP1.1 [SE]	12	673	8	7 (3)	1	0	2	0.53	4.24	2.65	0	2.40	*	1.38	*	1.87	*	7.51	*
PquPIP2.1	32	513	16	10	6	0	7	0.79	4.63	3.97	5	0.56		1.57	*	1.46		2.76	*
VsePIP2.1	46	627	10	7 (2)	2	1	17	0.89	2.72	2.27	47	0.56		0.20		0.37		- 7.48	
EfaPIP1.1 [NW]	104	459	12	10 (2)	2	0	19	0.87	3.27	2.30	59	1.11	*	0.83		1.11	*	- 4.20	*
EfaPIP1.1 [SE]	50	459	12	9 (2)	3	0	12	0.86	3.48	2.68	14	0.89		0.94		1.08		- 1.00	
EfaPIP1.2 [NW]	144	521	9	6 (1)	1	2	11	0.72	3.14	3.19	8	- 0.55		1.29		0.98		- 1.94	
EfaPIP1.2 [SE]	60	521	8	6 (1)	1	2	11	0.63	2.61	3.29	18	- 0.55		1.29	*	0.81		- 4.41	
EfaPIP2.1 [NW]	106	572	5	2 (1)	1	2	6	0.73	2.52	1.67	6	1.07	*	1.03		1.23		0.88	
EfaPIP2.1 [SE]	60	572	4	1 (1)	1	2	4	0.66	2.20	1.50	0	1.02		0.98		1.16		1.73	
EgrPIP2.1	194	671	14	9 (1)	2	3	10	0.23	1.21	2.40	0	- 1.24		1.53	*	0.58		- 2.11	

Legend: Analyses were performed taking recombination into account. N = sample size (number of sequences); L = amplicons length (base pairs); S = total number of segregating sites; S_I _= number of sites segregating in introns (number of indels in parentheses); S_S _= number of synonymous sites segregating in exons; S_A _= number of non-synonymous sites segregating in exons; A = number of observed haplotypes; H_d _= Nei's (1987) gene diversity computed on haplotype frequencies; *θ_π_*, *θ*_*w *_= estimates of population diversity parameter (4N_e_μ) from pairwise nucleotide differences and number of segregating sites, respectively (Tajima 1989); values multiplied by 1000; rho = population recombination parameter (4N_e_r); D, D*, F*, F_s_: standard neutral model statistics (see Materials and Methods). * = significant and the P = 0.05 threshold level. *n.b*. some significant values of *F_S _*are numerically negative but lie above the upper limit of the neutral confidence interval (see Additional File 1: Supplementary Table S1); they are therefore "statistically positive".

Haplotype (i.e. gametic phase) reconstruction was generally robust, with *P*-values for most genotypes higher than 0.90. The number of haplotypes varies greatly between species and populations with only one for *C. guianensis *population NW (PIP1.1) and 19 in *E. falcata *population NW (PIP1.1) in French Guiana. Overall, haplotypic diversity is large with values higher than 0.70 except in *C. guianensis *(*H_d _*equal to zero and 0.53 for PIP1.1 in NW and SE populations respectively) and *E. grandiflora *(*H_d _*= 0.23 for PIP2.1).

Two gene amplicons (VsePIP2.1 and EfaPIP1.1 population NW) have very high ρ values (Table 3), in spite of having levels of haplotypic diversity similar to those displayed by other amplicons, perhaps indicating large effective population sizes and/or past recombination with very divergent populations.

Tests for departures from the standard neutral equilibrium (Table 3) were performed taking into account recombination rates. The calculation of neutral confidence intervals for mutation-drift equilibrium statistics was performed at the most-likely values of *rho*, as a robustness test showed that the confidence interval limits of the statistics did not change substantially when values of *rho *at least 1000 times less likely than the most-likely value (ΔLOD ≤ 3) were used (see Additional File 1: Supplementary Table S1). In particular, as all significant tests are positive (see below), we were mostly interested in changes that shift upward the upper limit of neutral confidence intervals, which is the critical threshold for significance of positive values of the statistics. The only case for which a dramatic threshold change (> 10%) was observed is Tajima's *D *for *E. falcata *population SE at gene PIP1.2 (the upper limit of some values of *F_S _*is also modified, but this statistic is not biologically defined when positive). However, as shown in table 3, this statistic is not significant, and therefore changing *rho *estimates has no consequences for our results. For six amplicons/populations, mutation-drift equilibrium tests gave significant departures from neutral intervals: CguPIP1.1 (population SE), PquPIP1.1, EfaPIP1.1 (population NW), EfaPIP1.2 (population SE), EfaPIP2.1 (population NW) and EgrPIP2.1. Four amplicons/populations out of eleven did not reveal any departure from mutation-drift equilibrium (VsePIP2.1, EfaPIP1.1 (population SE), EfaPIP1.2 (population NW) and EfaPIP2.1 (population SE)). One amplicon/population combination (CguPIP1.1 population NW) could not be tested due to lack of polymorphism.

**Figure 2:**
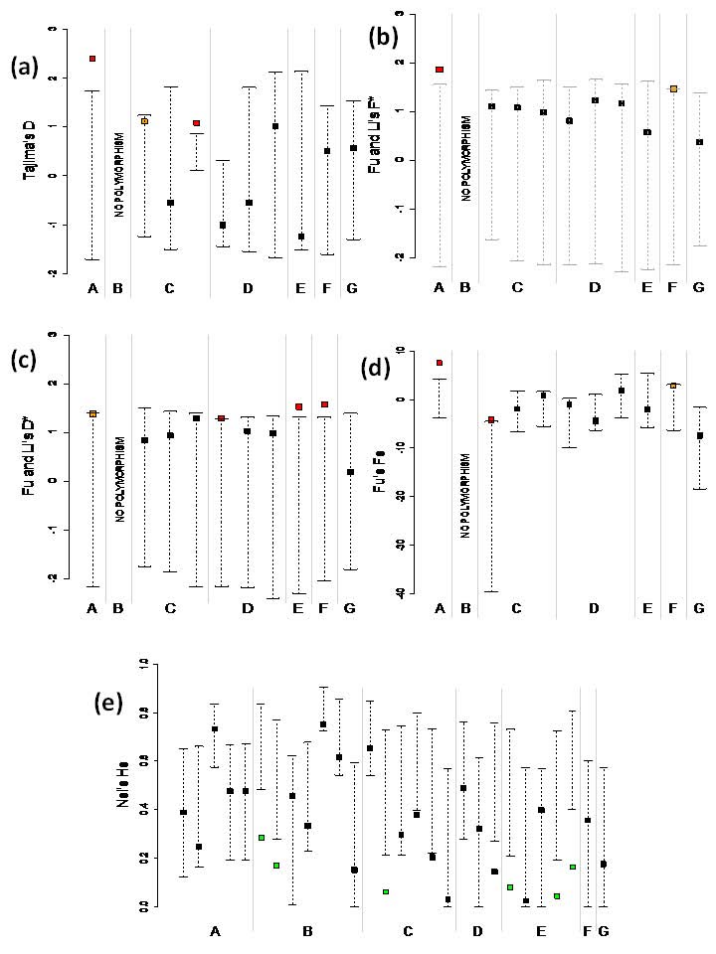
**Graphical representation of observed statistics and their neutral confidence intervals for each of the analysed genes**. **(a) **Tajima's *D *statistic; **(b) **Fu and Li's F* statistic; **(c) **Fu and Li's D* statistic; **(d) **Fu's *F_S _*statistic; **(e) **Nei's genetic diversity *H*. A, *Carapa guianensis *population SE; B, *Carapa guianensis *population NW; C, *Eperua falcata*, population NW; D, *Eperua falcata*, population SW; E, *Eperua grandiflora*; F, *Virola sebifera*; G, *Pachira quinata*. For each species and population, genes are the same as in Table 3. For C and D, genes appear in the order: EfaPIP1.1, EfaPIP1.2, EfaPIP2.1. Filled squares: observed values for each test (red, green = significant, *P *< 0.05; orange = marginally significant, 0.10 <*P *< 0.05). Dashed lines: neutral confidence intervals. Values exceeding the upper limit of the confidence interval (red) indicate balanced selection or bottleneck; values lower than the lower limit of the confidence interval (green) indicate negative selection or population expansion (in (e), green squares indicate significant values after Bonferroni correction). "No polymorphism": this population did not show any sequence variation for this gene.

Tests of selection on gene sequence data are summarised in Table 3 and Figure 2 (a-d). All significant tests show a departure from neutral allele frequency spectrum toward an excess of haplotypes at intermediate frequencies and/or deficit of rare variants (*n.b*. some values of *F_S _*are numerically negative but lie *above *the neutral confidence interval of the statistic after correction for historical recombination (see Additional File 1: Supplementary Table S1); thus they are effectively *positive *in terms of their statistical meaning). Although not all tests are significant, the global trend is towards positive and significant values for the test statistics. This indicates either the occurrence of a past bottleneck for most species or the ongoing action of balancing selection. No departure from (demographic) equilibrium towards population expansion or background selection was detected.

**Figure 3:**
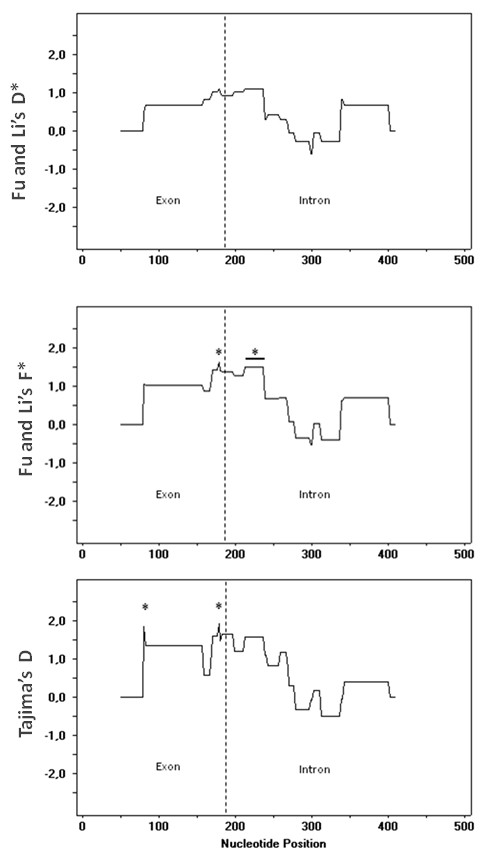
Sliding window of Fu and Li's F* and D* tests and Tajima's *D*, along EfaPIP1.1. Stars indicate the centre of windows showing a significant statistic. A horizontal bar indicates that several contiguous windows were significant.

For one gene (EfaPIP1.1), a sliding-window analysis of the mutation-drift equilibrium tests (Figure 3) shows that most positive and significant values are detected in windows centred at nucleotides 230-240, at or just downstream from the predicted intron splicing site. Two more sites display significant statistics within the exon.

**Figure 4:**
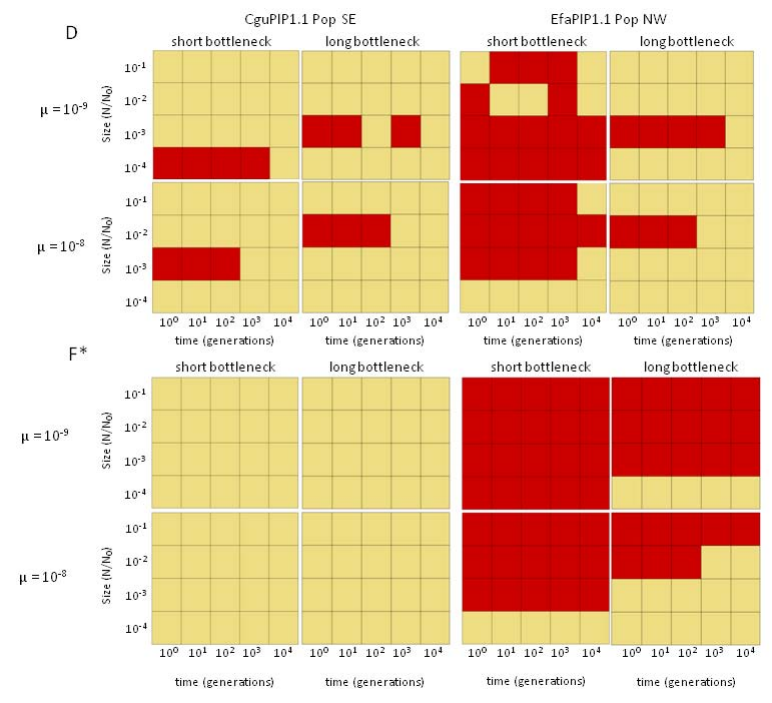
**Graphical representation of comparisons between observed test statistics and the 95% quantile of simulated test statistics**. Demographic scenarios were simulated with bottlenecks of variable age and strength and with low (μ = 10^-9^) and high (μ = 10^-8^) mutation rates. For all plots, *y*-axis: effective population size during the bottleneck, expressed as fraction of current population size; *x*-axis: approximate number of generations elapsed since the bottleneck. Lower left cells thus correspond to recent and strong bottlenecks, upper right cells to old and shallow bottlenecks. Dark cells correspond to scenarios compatible with the observed statistic (i.e. observed statistic is lower than the simulated 95% upper quantile). *Upper half*: tests for Tajima are D; *lower half*: tests for Fu and Li's F*. Left half: tests for CguPIP1.1 population SE; *right half*: tests for EfaPIP1.1 population NW.

To check whether the observed statistics were compatible with purely demographic events (i.e. bottlenecks in the case of positive tests), all significant mutation-drift equilibrium statistics were re-computed on simulated data sets having the same descriptive statistics as the observed data and having undergone bottlenecks of varying size, duration, and timing. Observed statistics were considered as compatible with a given demographic scenario if they fell within the 95% upper quantile of the values obtained on simulated data. The results of demographic simulations are illustrated in Figure 4 for Tajima's *D *and for Fu and Li's F*. The observed values of Tajima's *D *for *C. guianensis *gene CguPIP1.1 (population SE) (Figure 4 upper left pane) are only compatible with relatively recent and strong bottlenecks (in particular when the lower estimate of mutation rate is used). There is widespread compatibility with several demographic scenarios for the *E. falcata *gene EfaPIP1.1 (population NW) (Figure 4 upper right pane) when a short bottleneck is simulated, but not for simulations including a longer bottleneck. Simulations for Fu and Li's F* show that the observed statistic is entirely incompatible with any demographic scenario for *C. guianensis *(Figure 4 lower left pane), while most demographic scenarios are compatible with the observed value for EfaPIP1.1 population NW (Figure 4 lower right pane). Finally, all scenarios are compatible with the observed values for Fu and Li's D* (not shown) and with the significant Tajima's *D *test observed for EfaPIP2.1 (not shown). The significant departure from standard equilibrium observed for *P. quinata *(Fu and Li's D*) is also entirely compatible with demographic scenarios.

Results on demographic transitions, as detected by chloroplast SSRs, are shown in table 4 and visually summarised in Figure 2(e), and show global trends that tend to exclude bottlenecks: no species or population displayed signatures of population contraction. *C. guianensis, P. quinata *and *V. sebifera *did not display departures from mutation/drift equilibrium after Bonferroni correction, but the tests could only be performed on one locus for *V. sebifera *due to lack of polymorphism. However, the majority of tests tend to be negative, thus excluding a bottleneck signature. For *E. falcata *population NW and for *E. grandiflora*, values of test statistics against population bottlenecks were found at SSR loci concurrently with signatures of population contraction and/or balancing selection at PIP loci. *C. guianensis *population NW showed signatures contrary to bottlenecks at cpSSRs but was monomorphic for PIP sequences. *E. falcata *population SE also showed test statistics tending to exclude population bottlenecks, but no mutation-drift equilibrium test on gene amplicons was significant.

**Table 4:** Results of tests for demographic changes based on chloroplast SSR loci

Species/Population	N (e/c)	ccmp1	ccmp2	ccmp3	ccmp4	ccmp5	ccmp6	Ccmp7	ccmp10
*C. guianensis *[NW]	5 (0/0)	na	-4.187	-2.840	0.886	-1.062	-1.388	-1.049	-0.908
*C. guianensis *[SE]	7 (0/0)	na	0.010	M	-1.274	m	0.424	0.378	0.353
*P. quinata*	1 (0/0)	m	m	M	m	na	0.464	Na	m
*V. sebifera*	1 (0/0)	m	m	-0.517	m	m	m	M	m
*E. falcata *[NW]	6 (1/0)	m	-0.506	-3.109*	-1.340	na	-2.159	-2.084	-1.291
*E. falcata *[SE]	3 (1/0)	m	-0.243	M	0.136	na	m	-2.969	M
*E. grandiflora*	5 (3/0)	-2.925*	na	-1.288	0.941	na	-3.054*	-4.212*	Na

Legend: Ccmp1 through ccmp10: names of chloroplast SSR loci. The value of DH/sd, standardised departure from gene diversity (measured as hypothetical expected heterozygosity) expected under mutation/drift equilibrium, is reported. Positive values indicate population contraction; negative values indicate rejection of population bottleneck. Significance levels (after Bonferroni correction for multiple tests): *, *P *< 0.05. N = number of informative loci; e = number of loci indicating population expansion; c = number of loci indicating population contraction; m = monomorphic locus; na = locus which did not produce any PCR product.

## Discussion

The present study shows indications of the action of balancing selection on drought response-related genes in Neotropical tree species, although demographic changes in the populations, causing the same kind of departure from the standard neutral model, cannot be excluded, at least for some species (i.e. *P. quinata*, *E. grandiflora *and at least one population of *E. falcata*).

Extensive nucleotide diversity was found for six PIP gene fragments in five tropical tree species; the sequences obtained are useful for the detection of SNP polymorphisms, for genetic diversity analyses and for testing departures from expectations derived from the neutral theory of molecular evolution [27]. In one case (PIP2.1) the same primer pair was used to amplify two congeneric species (Table 2.b), providing a direct comparison between orthologs. For these two genes (EfaPIP2.1 and EgrPIP2.1, table 1) the level of polymorphism in *E. falcata *was lower than in *E. grandiflora*, but departures from mutation-drift equilibrium were detected in both species (Table 3), although not for the same statistics.

Standard neutral model tests on approximately half of the gene amplicons gave significant results. In genes and populations with significant departures from mutation-drift equilibrium the general trend is towards population size bottlenecks and/or balancing selection. However, sliding window analyses show that this trend does not apply uniformly to the entire sequence and that different regions of the genes may be subject to different processes. In one case (EfaPIP1.1; Figure 3), sites responsible for the positive and significant test statistics seem to be mostly restricted at the exon-intron boundary, possibly indicating selection on intron splicing sites or other regulatory functions by intronic sequences. Interestingly, when matched against small RNA databases, the intron fragment contained in EfPIP1.1 sequences is similar to immature *Arabidopsis thaliana *miRNA ath-MIR863 and to *Oryza sativa *miRNA osa-MIR420 (E-values of 0.048 and 0.064, respectively), indicating a putative functional role for the intron.

Two pieces of evidence favour balancing selection, rather than demography, as an explanation for the diversity patterns observed in this study, at least for a subset of the gene amplicons.

First, simulations of bottlenecks of variable sizes and ages, resulting in the same amounts of genetic diversity as observed in current populations, cannot produce statistics (Tajima's *D*, Fu and Li's F*) as high as those observed on real data for *C. guianensis *(Figure 4 left pane), unless moderate to strong and recent bottlenecks are assumed. D* values are however compatible with all demographic scenarios. For *E. falcata *(Figure 4 right pane), short bottlenecks are generally compatible with the observed Tajima's *D*, but not long bottlenecks (F* values are compatible with most demographic scenario in *E. falcata*, indicated by the fact that this statistic is not significant; table 3 and figure 2). As the species studied here were sampled in largely undisturbed portions of Neotropical forests, and are not known to have historically been exploited for timber, strong, short and very recent bottlenecks seem unlikely. However, bottlenecks hundreds of generations ago would be compatible with processes dating to the Late Pleistocene-Early Holocene, which would correspond to large-scale floristic changes in Neotropical forests [28]. The contrasting behaviour of D*, and F* and *D*, can be explained by what the statistics actually test and by the structure of our data. Both F* and *D *rely on estimation of the difference between an estimate of θ based on average pairwise sequence divergence and another estimate based on the number of segregating sites (singletons for F*, global estimate for D), whereas D* compares two estimates of numbers of segregating sites (common versus singleton). The former account for the depth of sequence divergence, whereas the latter does not. It appears that, at least in some cases, purely demographic scenarios cannot account for the excess sequence divergence observed in our data, while they can account for the observed number of segregating sites. This is an indication of exceedingly long genealogical branches in our data, which again favours balancing selection as opposed to bottlenecks. Moreover, all the tests applied have been shown to have similar power in the detection of recent bottlenecks [29], so that they are expected to give similar results under a purely demographic scenario.

Second, the tests on chloroplast SSRs generally refuted past bottlenecked populations, even if not all loci detect signatures of population expansion. Variability in the results from different loci is due to the fact that, if all loci describe the same evolutionary process, their statistics are multiple stochastic outcomes from the same probability function. As demography should influence all portions of the nuclear genome, as well as organellar genomes, in the same direction although with different sensitivity for the intensity and timing of demographic events [30], it is unlikely that events in opposing directions would be detected at different loci (recombination tends to smooth diversity estimates across genes, by reducing the variance of θ estimators [31]). Thus we can tentatively conclude that the departures from equilibrium at PIP loci are due to balancing selection. A caveat should be added that, as SSRs generally have different mutation rates than coding sequences, comparison of results from these two types of data may be misleading. In particular, chloroplast SSRs tend to mutate more quickly than nuclear, non-repeated sequences, although more slowly than nuclear SSRs [32], and the demographic events suggested by these markers may be more recent than the results of forces acting on sequence diversity. A further concern is that effective population size and demographic dynamics are not the same for chloroplast and nuclear loci, making the former, being haploid, more sensitive to demographic change. However, a recent demographic event detected in SSRs should also be detectable in sequences. Significant values for tests of departures from standard neutral equilibrium of opposite sign seem to support balancing selection on the expressed loci, while cases where demographic signatures are detected at chloroplast markers but not at PIP loci can be conservatively attributed to differences in marker sensitivity. Note, however, that this argument does not hold if the populations have undergone a bottleneck relatively far in the past (more than approximately 50 generations, the time beyond which the SSR-based methods used here cannot detect a bottleneck). In this case, fast-evolving SSRs may display the effects of the expansion following the bottleneck, while slow-evolving sequences may still display the effect of the bottleneck itself.

The observed departures from hypothetical selective neutrality in spite of a relative paucity of non-synonymous mutations is not surprising, given that regulatory sequences, potentially undergoing selection, may lie anywhere along gene sequences and that introns can have a function besides gene regulation. Among the gene fragments shown by mutation-drift equilibrium tests to putatively experience selection, VsePIP2.1 and EfaPIP1.1 do not contain non-synonymous mutations, and so, for these loci, selection may not be acting directly on the portion of protein-coding sequence analysed here. Selection may however affect other properties of the transcribed sequence, such as codon composition or intron functions. As PIP sequences are conserved among and within species [15] selection could rather act on regulatory regions. Alternatively, these polymorphism patterns may reflect selection on neighbouring sites, although the estimated population parameters suggest that recombination would quickly break down associations between selected sites and associated neutral sites, such that selection signatures may not extend beyond a few hundred base pairs from the site under selection. For genes that did not display any departure from expectations of the standard neutral model, full-length sequencing, including the promoter region, is advisable. In general, evolutionary patterns may diverge among different parts of a gene [33] and there is evidence of balancing selection in the promoter region of the *TFL1 *gene in *Arabidopsis thaliana *[34]. On the other hand, demographic events could be responsible for significant departures from the standard neutral model in the whole genome, including gene regions. Moreover, loci outside the sequenced region, but in linkage disequilibrium (LD) with it, may also affect the results of mutation-drift equilibrium tests. This possibility cannot be ruled out for the current data set, as the fragments are too short for testing the decay of LD with distance, although, in some species, population recombination rates are relatively high (Table 3), indicating that LD should rapidly fall to zero. This is generally the case for forest trees, where LD falls below 0.2 within 200-300 base pairs [10]). Therefore the main issue when observing significant mutation-drift equilibrium tests in genes is to discover whether selection or past demography underlie the observed levels of diversity.

For *C. guianensis *population SE, all tests for departure from mutation-drift equilibrium on locus PIP1.1 gave strongly significant results, while no chloroplast SSR marker did. This pattern is consistent with the maintenance of polymorphism by long-term balancing selection. It is interesting to note that the samples of population SE were collected from a hybridisation zone between *C. guianensis *and *C. procera*. Bayesian assignation methods, applied to independent loci (SSRs), show however that the sampled trees belong with a probability of 1 to *C. guianensis *populations [35], perhaps pointing to a stable and selectively advantageous introgression of *C. procera *genes into a *C. guianensis *genetic background. Actually, sequences of CguPIP1.1 obtained in pure *C. procera *stands show the same haplotype found in population SE of *C. guianensis *(Casalis *et al*. in preparation). If this hypothesis holds true, it may also explain the pattern of polymorphism observed for the two populations of this species: the NW population would display the "typical" status of the species, while the SE population, which would have undergone historical introgression, would carry an extra allele derived from the sister species *C. procera *(*n.b*. the taxonomic status of the latter species is under revision; P.M. Forget, personal communication). Further analyses of the diversity of PIP genes in the two species will test this hypothesis (M. Casalis et al. in prep.).

The results for PIP genes of *E. falcata *(the species for which the largest number of different genes was obtained) were divergent for the two populations. In summary (Table 3), population NW displays significant results for *D *and F* for genes PIP1.1 and PIP2.1, while population SE displays a significant test for gene PIP1.2 and the statistic D*. The results are almost perfectly complementary for the two populations: they show significant results for different genes *and *different statistics. Moreover, test statistics for population NW largely show incompatibility with demographic scenarios. As stated above, *D *and F* are more sensitive than D* to the extent of sequence divergence, and thus to balancing selection. Therefore, the simplest explanation for the observed pattern is that balancing selection is detected in population NW for two genes, while demography (*i.e*. a past population bottleneck) drives diversity patterns in population SE. The fact that chloroplast SSRs tend to support population *expansion*, rather than *bottleneck*, for population NW provides a further hint that the positive values of the mutation-drift equilibrium tests are due to selection rather than demography. Selection on the PIP1.1 gene would overcome the baseline demographic signal, which would provide *negative *statistics which are detected by SSRs (*n.b*. SSRs can only detect recent bottlenecks, while older ones may still affect sequence diversity). A similar argument can be applied to the only interspecific comparison, for gene PIP2.1 in the two *Eperua *species. Here, estimates of θ from pairwise sequence differences (θ_π_) are higher in *E. falcata *than in *E. grandiflora*, while estimates from haplotype diversity (θ_W_) are higher in *E. grandiflora *(Table 3). This apparent contradiction may be explained by the fact that *E. falcata *has a smaller number of more divergent haplotypes than *E. grandiflora *(A; table 3). Such results tend to favour different explanations for the diversity patterns in the two species: the historical preservation of highly divergent haplotypes in *E. falcata*, which is compatible with balancing selection (see above) and the presence of larger numbers of less differentiated haplotypes for *E. grandiflora*, which may be compatible with recovery from an old bottleneck or population expansion.

Finally, for *P. quinata*, departure from mutation-drift equilibrium was found for Fu and Li's D*, but the species displayed very little polymorphism at cpSSR loci, and therefore it was difficult to evaluate alternative hypotheses. The observed value of D* was, however, entirely compatible with demographic scenarios. Moreover, sample sizes per sampling site were relatively small for this species (see Methods) and, although tests for population differentiation were all non-significant, statistical power was low. Consequently, the departures from mutation-drift equilibrium may in this case be an artefact caused by hidden population structure. Large year to year variation (up to 100%) in overall rainfall and length of dry season may provide balancing selection, while extensive gene flow (bat pollination, seed dispersal by convection currents) may reduce population differentiation.

Among the cases described in this study, balancing selection may be the most common trend, but it is currently hard to outline why this should be the case. One possibility is that the sampled populations may be further structured along local ecological gradients at a smaller geographic scale, and that different environments favour alternative alleles, thus maintaining genetic diversity within populations. In this case, variation of ecological conditions at a spatial scale smaller than the size of populations may lead to the coexistence of different genetic optima in sub-structured populations, providing patterns that mimic balancing selection. As stated above, hidden population subdivision at loci responding to selective gradients can bias tests such as those applied here [36]. However, since we used PIP genes themselves to group individuals into non differentiated populations, this is likely to be a relatively minor problem in our data set. Alternatively, the wide variation in environmental conditions over time, both between seasons and over longer climatic cycles, may prevent selection from fixing a particular variant, explaining the balancing selection patterns. In general, although selective explanations of observed patterns of diversity should not be taken for granted unless solid evidence is provided, there is no reason either to assume that the most likely explanation should be a demographic one [37].

To test for the presence of selection, further studies could look for association between haplotype and adaptive traits related to water stress, as well as for selection in other candidate genes. Indeed, a large number of genomic or proteomic studies have identified candidate genes for water stress tolerance. For example dehydrin genes are up-regulated by drought stress (*BjDHN2 *and *BjDHN3 *in *Brassica juncea *[38]; *PgDhn1 *in *Picea glauca *[39]) and alcohol dehydrogenase (*Adh*) transcripts are induced by anoxia and hypoxia [40,41]. Contrasting the results for these genes with analyses of neutral loci to detect demographic events will also be fundamental to disentangle demographic versus adaptive interpretations of tests for departure from the standard neutral equilibrium model. As this is the first genomic study of gene sequences in non-plantation tropical trees, genomic resources that would allow such comparisons among different classes of genomic regions are not yet available.

Another limitation of our study is the length of the fragments analysed for each gene. Statistical tests, such as those we applied, gain more from increases in sequence length than from increases in sample size. Again, the complete lack of genomic resources for non-plantation tropical trees slows down the accumulation of sequence information, although isolation of larger sequences (Vedel *et al. *in prep.), as well as large-scale genome sequencing (Duret et al. *in prep*.) are under way. On the other hand, analysing larger sample sizes than generally suggested for this kind of studies was necessary, as the underlying distribution of genetic diversity and delimitation of populations was also unknown for these species. This information was necessary prior to the application of statistical tests. In spite of these limitations, the current data set allowed us to detect informative trends in sequence diversity. The results reported here should actually prove the feasibility of such research programmes, and are expected to prime more extensive investigations into the genomics of non-model tropical trees.

## Conclusion

The fragments of PIP (aquaporin) genes analysed here have revealed large variability and potentially strong signatures of past population-genetic events, in some cases with patterns that vary along the gene. Besides being the first report on molecular diversity in genes of ecologically relevant species of one of the most diverse biomes on Earth and on non-plantation tropical trees, the results presented here, if confirmed by further studies, may convey some interesting messages. First, high levels of diversity at PIP genes may be maintained by selective mechanisms - either *sensu strictu *balancing selection or divergent selection at a local scale, which mimics the effects of balancing selection. Second, this trend appears to be common to a diverse array of species. Extended characterisation of these loci is likely to reveal more details on the processes shaping their diversity and to provide information on the link between genetic diversity and ecological conditions. More generally, systematic characterisation of candidate genes may lead to a more complete picture of the way genotypes interact with their environment in tropical forests and other ecosystems. We are convinced that this is a necessary step towards the consolidation of ecological genetic understanding of tropical ecology. At the same time, the suggested presence of balancing selection seems to fit well in the greater picture of tropical ecosystems: the maintenance of gene diversity by selection at the species level may be another facet of the many mechanisms proposed to explain the high levels of biological diversity observed in the tropics. The extant genetic diversity, and its maintenance by selection, may indicate that these species harbour the adaptive potential to cope with future, expected climate change. This will be particularly crucial if tropical rainforests undergo increased droughts that threaten [5] to severely affect their productivity and therefore their survival.

## Methods

### Sampling

Five Neotropical tree species from four different families were selected for covering varying geographic and environmental ranges. *Carapa guianensis*, (Meliaceae), *Pachira quinata *(Bombacaceae) and *Virola sebifera *(Myristicaceae) display a continental distribution whereas *Eperua falcata *and *E. grandiflora *are endemic to the Guiana shield. All species except *Pachira quinata*, display preferences for wet, flooded environments (see table 5). *Pachira quinata *was sampled in three countries (Colombia, Costa Rica and Honduras) across a range of soil (loam to seasonally flooded vertisols) and rainfall (1200 to 3500 mm/year, 3-7 month long dry season) conditions, whereas the other four species were sampled in French Guiana (see Table 5). French Guiana is characterised by annual rainfall varying between 5000 and 2500 mm/year, and by strong seasonality in rainfall (June to December dry season characterised by long and severe dry spells). In French Guiana 5-10 samples per species were collected at sites separated by at least 10 km along a North-West to South-East transect, that spans the rainfall gradient. The total number of samples per species depends on the frequency of occurrence of each species at each site. At each site, samples were collected so that no tree was farther apart from the next sampled tree than the average gene dispersal distance for the species (or congeneric species when information on the species was not available) [42]. This ensures sampling of panmictic populations within sites.

**Table 5:** Sampling sites and species characteristics

Species	Genus range	Species range	Habitat preferences	Sampling sites and sample size (number of individuals sampled)				
				Colombia 74°50' W 11°00' N	Costa Rica 85˚09' - 85˚19' W 09˚35' - 10˚23' N	Honduras 87˚27' W 13˚16' N	Northwest French Guiana 52°21' - 54°08'W 4°61' - 5°29'N	Southeast French Guiana 52°12' - 53°12'W 3°38' - 4°41'N
				
*Carapa guianensis*	Americas, Africa	Continental	wet,flooded/ canopy gaps				29	6
*Pachira quinata*	Americas, Africa	Continental	wet,seasonally flooded to dry/forest to open	4	8	4		
*Virola sebifera*	Americas	Continental	wet, flooded/ forest				23	
*Eperua falcata*	Regional (Guiana shield)	regional (Guiana shield)	wet, flooded/ forest				72	30
*Eperua grandiflora*	Regional (Guiana shield)	regional (Guiana shield)	wet, drained/ forest				96	

### DNA extraction

For all species except *Pachira quinata*, total genomic DNA was extracted from cambium or leaf tissues following a CTAB method adapted from [43] and [44], starting from approximately 1 cm² of silica gel-dried tissue. DNA quality was analysed by spectrophotometry at 260 nm and 280 nm or by agarose gel electrophoresis. For *P. quinata*, Qiagen DNeasy 96 Plant Kit (69181) was used to extract genomic DNA from embryos.

### Universal primer design, PCR amplification and DNA sequencing

**Figure 5:**
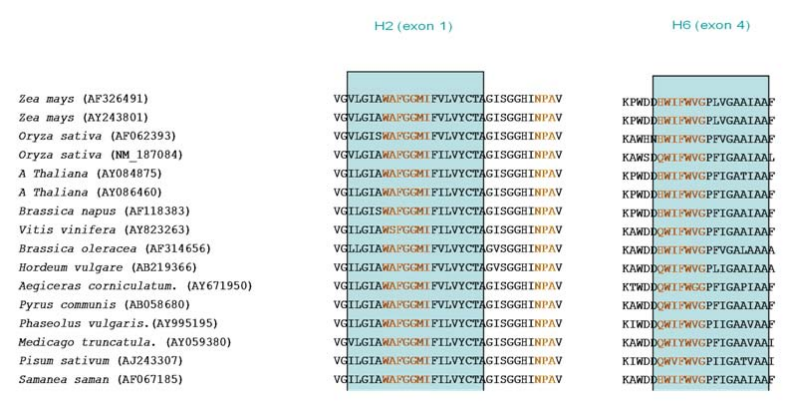
**PIP1 - PIP2 conserved regions chosen based on CLUSTALW alignments.** Blue rectangles represent transmembrane helices two and six, in which the universal primers were chosen (amino-acid sequences in red) (only a subsample of aligned sequences is shown).

To isolate PIP genes from tropical tree species, all nucleic and amino-acids sequences of Dicotyledonous PIP1 and PIP2 subfamilies available in GenBank were aligned with CLUSTALW. Degenerated, universal primers were designed in the most conserved regions (Figure 5, table 2). To isolate PCR fragments corresponding to the selected loci, two alternative strategies were used, in both cases using degenerate primers for the amplification of fragments corresponding to genes in the subfamilies PIP1 and PIP2. (a) For *Eperua falcata*, RNA was isolated using the protocol described in [45] and cDNA was obtained using Lambda-ZAP-cDNA Synthesis Kit (Stratagene); (b) for *Carapa guianensis, Pachira quinata, Virola sebifera*, degenerate PCRs were carried out on genomic DNA. As a control, the latter protocol was applied to *E. falcata*, to check that the two methods provided similar results. Details of PCR and sequencing conditions are reported in Additional file 2: Supplementary Methods 1(a).

### Sequence analysis and specific primer design

Base calling and contig assembly were done using CodonCode Aligner v2.0.1 (Codoncode Corporation, Dedham, MA). For each species, contigs were named according to their closest match (either PIP1 or PIP2) in the TAIR database [26], followed by contig number. The partition of genomic fragments in exons and introns (Figure 1) was obtained by alignment of the genomic fragments with publicly available mRNA sequences. To infer whether introns contained any conserved motif, that may undergo natural selection, homology between intron sequences and small RNA families was checked by matching the former to the latter on the Sanger Centre's microRNA database [46].

Sequence information was used for the development of gene-specific and species-specific primer pairs (Table 2, Figure 1). For the identification of priming regions 96 clones were sequenced on both strands for *E. falcata*, 46 for *C. guianensis *and *P. quinata *and 22 for *V. sebifera*. The sequences retrieved in *E. falcata *from cDNA and genomic DNA PCRs closely matched (not shown), thus confirming the equivalence between the two strategies for the isolation of fragments of coding regions. The PCR products obtained for each species were expected to contain a mix of fragments from several members of the PIP subfamily, and therefore clones corresponding to different genes had to be sorted prior to the design of specific primers. To do this, sequences were aligned and grouped into clusters using ClustalW [47]. Clusters contained closely related sequencing runs (with less than 5% sequence divergence), thus allowing for sequencing errors; each cluster was considered as representing a different gene. Cluster consensus sequences were aligned and the most divergent regions were identified and used for the subsequent step. Species- and locus-specific primers (tables 1 and 2) were designed in the outermost regions that granted sufficient divergence among contigs within each species to obtain the largest possible fragments with the best specificity. Primer pairs were used to amplify genomic DNA from 8-16 samples per species. Primers pairs that produced multiple PCR products were discarded. Those that produced a single product were sequenced. The sequence traces of a subset of primer pairs showed at least two overlapping sequences, possibly indicating the co-amplification of more than one gene; these primer pairs were also discarded. Details on protocols for gene- and species- specific PCR are provided in Additional file 2: Supplementary Methods 1(b).

### DNA polymorphism, population structure and demographic processes

Since the DNA samples were diploid, the identification of haplotypes (*i.e*. sequence variants) was ambiguous where more than one SNP was present and heterozygote individuals were observed. Diploid sequences were treated using Haplotyper [48] to produce two haploid sequences per individual. Insertions-deletions ("indels") were coded like SNPs: each gap, irrespective of its length, was replaced by a nucleotide producing a SNP to treat indels in subsequent analyses. Indel inference in heterozygote samples was performed based on the comparison of sequences obtained from the two strands and by applying the "Split heterozygote indels" function in CodonCode Aligner.

Population sub-structuring introduces biases in the outcome of mutation-drift equilibrium tests, and therefore the latter must be applied exclusively to panmictic, Wright-Fisher populations (although some tests are robust to population structure). Therefore, the groups of samples obtained for each sampling site were tested for genetic differentiation - based on haplotypes at PIP genes - and lumped together when differentiation could not be detected. For *E. falcata *and *C. guianensis*, sampling sites turned out to belong to two clusters (identified as NW and SE populations in tables 3 and 4) and therefore standard neutral equilibrium model tests were performed separately for each population.

Analyses of sequence data were performed using DnaSP v. 4.10.9 [49]. Nucleotide diversity was estimated by Watterson's θ_w _[50] and π, the average number of pairwise nucleotide differences among sequences in a sample [51].

Coalescent simulations with DnaSP were performed with recombination, because the accumulation of historical recombination events influences patterns of sequence diversity even on very short genetic distances. Within-amplicon recombination rate of gene fragments was estimated by LDhat v2.1 [52]; most-likely values of *rho *and their confidence interval (i.e. the interval of values with a ΔLOD no larger than 3 from the most likely value) were used to test the robustness of standard neutral equilibrium statistics (see Results) to variations of *rho*. That is, neutral confidence intervals for each statistic were obtained and compared for the most likely value of *rho *and for two estimates, one on each side of the most likely estimate, the probability of which is 1000-fold lower than the probability of the most likely estimate.

Tajima's *D *[53], Fu and Li's D* and F* [54] and Fu's *F_s _*[55] tests were computed to identify departures from the standard neutral model of evolution. All these tests are based on the comparison of observed levels of DNA sequence diversity obtained from different estimators, which, under neutral conditions of a population with stable effective size and in the absence of selection, estimate the population diversity parameter *theta*. Departures from the standard neutral equilibrium model affect the various estimators differently, causing their (standardised) difference to be non-zero. Tajima's *D*-statistic was computed for each locus and reflects the difference between π and θ_W_. Fu and Li's tests (D* and F*) are based on the distribution of mutations in the genealogy and compare the number of "old" and "new" mutations. The *F*_s _test, based on the haplotype (gene) frequency distribution, was also calculated. These tests were preferred over those requiring comparisons to an out-group, due to lack of genomic information on closely related species and the difficulty of correctly identifying orthologs in multiple-gene families such as PIPs. All these statistics were also estimated within each sequence by a sliding-window method. Test statistics were recomputed on windows of one-hundred base pairs length with a step size of two using the function implemented in DnaSP [49].

Since both selection and variations in effective population size can affect the statistics in similar ways, there is no way to deduce which evolutionary force is acting on the populations based on the simple observation of departure from mutation-drift equilibrium in a given direction. We applied two independent strategies to split the effects of different evolutionary forces on the statistics. (1) To test whether purely demographic events were sufficient to explain the observed values of the statistics, simulations were performed using the MS program [56]. Samples of the same size and diversity parameters (theta, population recombination rate, number of segregating sites) as the observed populations were simulated. Simulations were performed assuming a mutation rate per generation per site of μ = 10^-8 ^and μ = 10^-9^; approximate estimations of effective population sizes were carried out as in [16]. Bottleneck events were simulated [20] for a reduction to a population size between 1/10 and 1/1000 of current estimated population sizes, having occurred between one and ten thousand generations before present, and having lasted for 10 or 100 generations (after which pre-bottleneck population size is instantly restored). These parameters were chosen to provide scenarios with expected positive values for the estimated statistics, as the only significant observed values of *D*, D* and F* (see results) were positive. Simulations were not performed for *F_S _*as this statistic has a clear meaning only when negative [55], whereas only positive significant values were obtained. One-hundred samples were simulated for each combination of bottleneck size, duration and age, and the 95% upper quantile was computed for all statistics for each combination of parameters. Tajima's *D *was estimated directly with the MS program, whereas D* and F* were computed on the simulated data sets with a suite of R [57] routines specifically designed for this purpose, and available from the corresponding author. The observed values were then compared to the 95% upper quantile of the distribution obtained for each combination of parameters. When the observed value fell below the simulated 95% threshold, it was considered as compatible with the demographic scenario defined by the parameters. In this way, we have devised a strictly conservative test for the detection of balancing selection: the presence of selection is assumed only when no past change in effective population size can be claimed to be responsible for the observed departure from standard neutral equilibrium. (2) The same samples analysed for sequence diversity were also genotyped at eight chloroplast loci using universal primer pairs (ccmp1, ccmp2, ccmp3, ccmp4, ccmp5, ccmp6, ccmp7, ccmp10 [58]). The patterns of diversity obtained on these eight loci were analysed with the "sign test" method included in the BOTTLENECK software package [59] to test for (recent) demographic events in the populations, that may be detected by tests of selection on sequences and thus confound the results. The Stepwise Mutation Model and 1000 replications were used for these tests. The results from chloroplast microsatellites were used as the neutral reference, for which departures from the equilibrium can only be caused by demographic events. Departures from neutral equilibrium, observed on PIP sequences, were compared to results obtained on cpSSR markers to make inferences on the processes that shaped diversity patterns at gene sequences. For this analysis the loci, although fully linked, were tested independently because combining multiple sites, each mutating independently, into a synthetic genotype may introduce a bias on significance thresholds, which are based on independent loci, and not a linear combination of repeat lengths of multiple loci. We are unaware of mutational models that actually take into account linear combinations of SSR loci. On the other hand, no multi-locus test was performed, as they also assume independence. We instead applied a Bonferroni correction to significance thresholds for each of the SSR loci, considered as multiple realisations of the same expected statistical distribution (i.e. the expected frequency spectrum of SSR loci at mutation-drift equilibrium, conditioned on observed number of alleles). Since the method is not affected by departure from Hardy-Weinberg equilibrium [60] it can be applied to haploid data, provided they are from a panmictic population.

## Authors' contributions

DB, IS, GGV contributed to experimental conception and setup; DA, CSS, IS, DB, PR contributed to sampling strategy choice and sampling; DA, AB, PR, GGV contributed to sequence and marker data collection; DA, AB, LB, IS, GGV contributed to gene isolation and choice of resequencing strategies; DA, IS, CSS contributed to data analyses. All authors read and approved the final manuscript.

## Supplementary Material

Additional File 1 **Supplementary table S1**. Neutral confidence intervals for mutation-drift equilibrium statistics as a function of *rho *for each Gene/population Click here for file

Additional file 2 **Supplementary methods 1**. **(a) *PCR and sequencing conditions for the isolation of gene sequences *and (b) *Conditions for Specific PCR amplifications***. Click here for file
