# Supplementary material for: Aquaporins in the wild: natural genetic diversity and selective pressure in the PIP gene family in five Neotropical tree species
Source: BMC Evol Biol. 2010 Jun 29;10:202. doi: 10.1186/1471-2148-10-202 (PMC2906476; doi:10.1186/1471-2148-10-202)
Supplement: Additional File 1 — Supplementary table S1. Neutral confidence intervals for mutation-drift equilibrium statistics as a function of rho for each Gene/population [file 1471-2148-10-202-S1.DOC]

Supplementary Table S1.  **Neutral confidence intervals for mutation-drift equilibrium statistics as a function of *rho* for each Gene/population.**

| Gene/Population | *rho* value | *D* | *D** | *F** | *Fs* |
| --- | --- | --- | --- | --- | --- |
| CguPIP1.1 pop1 | *rho* 1 = 0 | [-1.77 ; 1.75] | [-2.11 ; 1.41] | [-2.40 ; 1.54] | [-3.52 ; 4.72] |
|  | *rho* 2 = 0 | [-1.77 ; 1.75] | [-2.11 ; 1.41] | [-2.40 ; 1.54] | [-3.52 ; 4.72] |
|  | *rho* 3 = 0 | [-1.77 ; 1.75] | [-2.11 ; 1.41] | [-2.40 ; 1.54] | [-3.52 ; 4.72] |
| CguPIP1.1 pop2 | - | - | - | - | - |
| PquPIP2.1 | *rho* 1 = 1 | [-1.61 ; 1.67] | [-2.29 ; 1.39] | [-2.25 ; 1.59] | [5.33 ; 4.45] |
|  | *rho* 2 = 2 | [-1.67 ; 1.73] | [-2.25 ; 1.36] | [-2.39 ; 1.55] | [-5.92 ; 3.55] |
|  | *rho* 3 = 6 | [-1.57 ; 1.39] | [-1.88 ; 1.34] | [-1.95 ; 1.50] | [-7.21 ; 2.11] |
| VsePIP2.1 | *rho* 1 = 18 | [-1.32 ; 1.46] | [-2.20 ; 1.44] | [-1.87 ; 1.45] | [-9.93 ; -0.31] |
|  | *rho* 2 = 47 | [-1.30 ; 1.54] | [-1.81 ; 1.35] | [-1.69 ; 1.53] | [-18.42 ; -1.54] |
|  | *rho* 3 = 85 | [-1.30 ; 1.39] | [-1.60 ; 1.40] | [-1.88 ; 1.44] | [-28.29 ; -1.98] |
| EfaPIP1.1 pop1 | *rho* 1 = 41 | [-1.23 ; 1.32] | [-2.01 ; 1.53] | [-1.98 ; 1.46] | [-27.75 ; 3.29] |
|  | *rho* 2 = 59 | [-1.23 ; 1.48] | [-1.69 ; 1.46] | [-1.54 ; 1.43] | [-41.38 ; -4.56] |
|  | *rho* 3 = 98 | [-1.18 ; 1.28] | [-1.73 ; 1.50] | [-1.69 ; 1.37] | [-56.18 ; -6.68] |
| EfaPIP1.1 pop2 | *rho* 1 = 8 | [-1.45 ; 1.60] | [-1.85 ; 1.44] | [-2.04 ; 1.49] | [-7.37 ; 1.49] |
|  | *rho* 2 = 14 | [-1.44 ; 1.46] | [-1.90 ; 1.35] | [-1.96 ; 1.43] | [-9.95 ; 0.32] |
|  | *rho* 3 = 27 | [-1.40 ; 1.39] | [-1.76 ; 1.43] | [-1.69 ; 1.49] | [-14.80 ; -1.49] |
| EfaPIP1.2 pop1 | *rho* 1 = 3 | [-1.48 ; 1.83] | [-2.40 ; 1.29] | [-2.42 ; 1.64] | [-5.57 ; 2.76] |
|  | *rho* 2 = 8 | [-1.51 ; 1.82] | [-2.38 ; 1.29] | [-2.03 ; 1.61] | [-6.74 ; 1.83] |
|  | *rho* 3 = 26 | [-1.42 ; 1.70] | [-2.08 ; 1.29] | [-2.12 ; 1.46] | [-12.16 ; 0.20] |
| EfaPIP1.2 pop2 | *rho* 1 = 2 | [1.63 ; 2.02] | [-2.08 ; 1.34] | [-2.05 ; 1.63] | [-4.22 ; 3.52] |
|  | *rho* 2 = 18 | [-1.55 ; 1.81] | [-2.40 ; 1.34] | [-1.97 ; 1.55] | [-6.36 ; 1.07] |
|  | *rho* 3 = 62 | [-1.47 ; 1.62] | [-2.40 ; 1.29] | [-2.08 ; 1.46] | [-13.95 ; 0.29] |
| EfaPIP2.1 pop1 | *rho* 1 = 1 | [0.09 ; 0.82] | [-2.17 ; 1.41] | [-2.26 ; 1.57] | [-5.13 ; 3.77] |
|  | *rho* 2 = 6 | [0.09 ; 0.86] | [-2.17 ;1.36] | [-2.08 ; 1.57] | [-5.55 ; 1.64] |
|  | *rho* 3 = 23 | [0.09 ; 0.90] | [-2.17 ; 1.31] | [-2.07 ; 1.60] | [-9.45 ; 0.71] |
| EfaPIP2.1 pop2 | *rho* 1 = 0 | [-1.67 ; 2.13] | [-2.61 ; 1.34] | [-2.52 ; 1.53] | [-3.73 ; 5.20] |
|  | *rho* 2 = 0 | [-1.67 ; 2.13] | [-2.61 ; 1.34] | [-2.52 ; 1.53] | [-3.73 ; 5.20] |
|  | *rho* 3 = 5 | [-1.50 ; 1.82] | [-2.40 ; 1.34] | [-2.40 ; 1.57] | [-4.48 ; 2.22] |
| EgrPIP2.1 | *rho* 1 = 0 | [-1.52 ; 2.14] | [-2.44 . 1.33] | [-2.41 ; 1.66] | [-5.77 ; 5.40] |
|  | *rho* 2 = 0 | [-1.52 ; 2.14] | [-2.44 . 1.33] | [-2.41 ; 1.66] | [-5.77 ; 5.40] |
|  | *rho* 3 = 1 | [-1.52 ; 2.15] | [-2.44 ; 1.33] | [-2.28 ; 1.66] | [-5.84 ; 4.34] |

*rho*1 = lower limit of the credible interval of *rho* (smallest value of *rho* having a ΔLOD≤3 from the most likely value); *rho*2 = most-likely *rho* value; *rho*3 = upper limit of the credible interval of *rho* (largest value of *rho* having a ΔLOD≤3 from the most likely value).
