# Supplementary material for: Aquaporins in the wild: natural genetic diversity and selective pressure in the PIP gene family in five Neotropical tree species
Source: BMC Evol Biol. 2010 Jun 29;10:202. doi: 10.1186/1471-2148-10-202 (PMC2906476; doi:10.1186/1471-2148-10-202)
Supplement: Additional file 2 — Supplementary methods 1. (a) PCR and sequencing conditions for the isolation of gene sequences and (b) Conditions for Specific PCR amplifications. [file 1471-2148-10-202-S2.DOC]

## Supplementary Methods 1

#### (a) PCR and sequencing conditions for the isolation of gene sequences.

(a) *Eperua falcata*

PCR was carried out in a 25 µL-volume containing 5µl cDNA, 1x *Taq* buffer, 1.6 mM MgCl_2_, 0.24 mM of each dNTP, 1 U Taq polymerase (all products from Invitrogen) and 0.6 µM of each primer. An initial denaturation at 94°C for 5 min was followed by 35 cycles of (45 s at 94°C, 45 s at 64°C and 45 s at 72°C) and a final extension of 5 min at 72°C after the cycles. To isolate the various PIP sequences, PCR products were cloned into pGEM®-T vector (Promega) and PCRs performed on 48 colonies with the same protocol as described above, but with a lower annealing temperature (60°C). PCR products were cleaned-up with EXOSAP-IT (USB Corporation). Sequencing reactions were performed with BigDye® Terminator v3.1 cycle sequencing kit (Applied Biosystems) in a total volume of 10 µl containing 1.5 µl of Big Dye, 1.5 µl of Buffer, 2 µl of 2 µM primer, 4 µl of cleaned-up PCR product and 1µl of milli-Q water. All fragments were sequenced in both directions. Sequencing reactions were then purified on Sephadex columns (Millipore) and sequence data obtained on an ABI 3130xl capillary sequencer (Applied Biosystems).

(b) *Carapa guianensis, Pachira quinata, Virola sebifera*

Genomic DNA was extracted with the Invisorb Spin Plant kit (Invitek); universal primers (table 2.a) were used for PCR in a total volume of 12.5 μL, containing 10 ng of genomic DNA, 1×PCR buffer (Promega, 1,5 mM MgCl_2_ final concentration), 0,2 μM each primer, 0,2 mM each dNTP, 1 U Go Taq polymerase (Promega), 0.8% BSA. The reaction was performed with the following thermal profile: 4 min at 94°C; 35 cycles of (30 s at 94°C, 30 s at 50°C, 40 s at 72°C); and a final extension of 7 min at 72°C. Following the PCR, the amplified products were cloned into TOPO vector (Invitrogen) and then sequenced from both ends using a MegaBace 1000 capillary sequencer (Amersham), with the same protocol as above.

#### (b) Conditions for Specific PCR amplifications

Specific primers were applied to the amplification of specific genomic DNA; primers drawn from *E. falcata* were also tested in the congeneric *Eperua grandiflora*. Genomic DNA of *E. falcata*, *E. grandiflora* and *C. guianensis* was amplified with the following PCR conditions for all primer pairs: PCRs were carried out in a 15 µL-volume containing 15 ng of DNA, 1x *Taq* buffer, 3 mM MgCl_2_, 0.25 mM of each dNTP, 0.6 U Taq polymerase (all products from New England Biolabs) and 0.5 µM of each primer. An initial denaturation at 94°C for 4 min was followed by 35 cycles of (45 s at 94°C, 30 s at the annealing temperature shown in Table 2.a and 1 min 30 s at 72°C) and a 10 min final extension at 72°C after the cycles. For *V. sebifera*, PCR reactions were performed in a 12.5 µL reaction volume containing 10 ng of DNA, 1×PCR buffer (Promega), 0.2 µM each primer, 0.2 mM each dNTP, 2.5 mM MgCl_2_, 1 U Go Taq polymerase (Promega) and 0.8% BSA, using the following thermal profile: initial denaturation at 95°C for 5 min, 30 cycles of (30 s at 95°C, 30 s at 59°C, 30 s at 72°C); final extension at 72°C for 7 min. For *P. quinata*, PCRs were performed in a total volume of 10µL reaction containing 0.25mM BSA, 0.6uL 0.15mM dNTPs, 2mM MgCl_2_, 0.2mM forward and reverse primers, 0.5U Taq (Yorkshire Biosciences A2002 YB-TAQ DNA Polymerase). The following thermal profile was applied: 3 min at 94 °C, 30 cycles of (30 s at 94 °C, 30 s at 64°C, 1 min at 72 °C), final extension at 72 °C for 10 min.
